# Effect of nitric oxide donor and plasma volume expansion on pregnancies with early onset fetal growth restriction: a randomized controlled trial

**DOI:** 10.1007/s00404-025-07988-7

**Published:** 2025-03-24

**Authors:** Maya Mahmoud Abdelrazek, Mai Abdelfattah Ahmed, Mohamed Elmandooh Mohamed Ibrahim

**Affiliations:** Obstetrics and Gynecology Department, Faculty of Medicine, Ain Shams University, Cairo, Egypt

**Keywords:** Nitroglycerine, Fetal growth, Estimated fetal weight, Umbilical artery doppler, Amniotic fluid volume

## Abstract

**Purpose:**

To evaluate the effect of nitric oxide (NO) donor, in combination with plasma volume expansion, on both fetal and maternal outcomes in pregnancies complicated by early-onset fetal growth restriction (FGR).

**Methods:**

A total of 40 pregnant women diagnosed with early onset FGR were recruited from Ain Shams University Maternity Hospital between June 2023 to December 2023. The patients were randomly assigned into two groups, 20 in each group. Group A received Nitroderm TTS ^®^ 5 mg for 12 h daily with plasma volume expansion (PVE) in the form of 2.5 L of water per day. Group B represented the control group. The primary endpoint of the study, assessed after 2 weeks of treatment initiation, focused on fetal growth parameters as the primary outcome. In addition, amniotic fluid volume, umbilical artery Doppler changes, development of fetal complications, maternal vital signs, and any side effects, were recorded. At the time of delivery, the following also documented: timing, mode, and interval to delivery, along with neonatal outcomes.

**Results:**

Group A exhibit statistically significant enhancement in fetal growth compared to Group B in terms of estimated fetal weight, abdominal circumference, head circumference, biparietal diameter, femur length, amniotic fluid volume, and umbilical artery pulsatility index. Furthermore, Group A demonstrated more favorable outcomes in terms of gestational age at delivery, interval to delivery, birth weight, APGAR score and rates of NICU admission.

**Conclusion:**

The combination of NO donors and PVE has shown promising results in enhancing fetal growth and extending gestation. This study adds to the existing body of evidence supporting the effectiveness of NO donor therapy when used in conjunction with fluid management for managing FGR. Nonetheless, additional research is essential to validate these results and refine the treatment strategy for optimal outcomes in affected pregnancies.

## What does this study add to the clinical work

**Table:** 

This study demonstrates that combining nitric oxide donors with plasma volume expansion significantly enhances fetal growth and Doppler indices, ultimately prolonging gestation in pregnancies affected by early-onset fetal growth restriction and improving neonatal outcomes.	

## Introduction

Fetal growth restriction (FGR) is a condition where there is a pathological restriction of the fetal genetic growth potential [1]. It is commonly diagnosed based on an estimated fetal weight (EFW) or abdominal circumference (AC) less than the 10th percentile for gestational age [2]. While some FGR fetuses are small for gestational age (SGA), the likelihood of FGR is higher in fetuses that has EFW below 3rd percentile, which is associated with higher risks of fetal compromise, particularly when accompanied by abnormal Doppler indices and/or reduced amniotic fluid volume [1, 3].

FGR is broadly categorized into early- and late- onset depending on the gestational age at diagnosis, occurring before or after 32 weeks. Early-onset FGR, which is more severe, is often linked to placental insufficiency and results in worse perinatal outcomes, including preterm birth, preeclampsia, and increased morbidity and mortality [1, 3–6]. In contrast, late-onset FGR, though more common, is associated with milder perinatal risks but may still impact neurodevelopment [7].

Studies have shown that pregnancies complicated by FGR often exhibit cardiovascular maladaptation, including reduced maternal intravascular volume expansion, lower stroke volume (SV), and cardiac output (CO), along with increased maternal vascular resistance [8]. This hemodynamic imbalance suggests a potential role for therapeutic interventions aimed at improving uteroplacental blood flow.

Nitric oxide (NO), a vasodilatory molecule produced by endothelial cells, plays a critical role in regulating placental vascular tone and development [9]. It reduces vascular resistance in the uteroplacental and fetoplacental circulations, which may have therapeutic implications for managing FGR [10]. NO donors, when combined with plasma volume expansion (PVE), could improve maternal cardiovascular function and uteroplacental perfusion, potentially enhancing fetal growth in pregnancies complicated by FGR [11].

Glyceryl trinitrate, a well-known NO donor, has shown efficacy in improving Doppler indices and maternal blood pressure. However, side effects such as headache and the development of tolerance limit its use during pregnancy [12]. While previous studies have investigated the cardiovascular effects of NO and PVE separately, but little is known about their combined effect on maternal hemodynamics and fetal outcomes in severe early-onset FGR.

This study aims to evaluate the impact of combining NO donors with PVE on pregnancies affected by early-onset FGR. We hypothesize that this approach can improve uteroplacental blood flow, enhance fetal growth, and improve Doppler indices while minimizing maternal side effects.

## Material and methods

### Study population and setting

This randomized controlled study included 40 pregnant women diagnosed with early onset FGR at a tertiary University Hospital, Ain Shams University, between June 2023 and December 2023. Before the study began, ethical approval was granted by the faculty of Medicine Ain Shams University Research Ethics Committee (FMASU REC) under the reference number FMASU MS 533/2023 and all subjects provided written informed consent.

Included women with a singleton pregnancy, diagnosed with early-onset FGR based on the criteria proposed by an international Delphi consensus [3]. This diagnosis included gestational age < 32 weeks, the absence of congenital anomalies, and either Abdominal Circumference/Estimated Fetal Weight (AC/EFW) < 3rd percentile or umbilical artery Doppler reveled absent end diastolic flow (UA-AEDF). Alternatively, diagnosis could be based on a combination of AC/EFW < 10th percentile with uterine artery-pulsatility index (UtA-PI) > 95th percentile or umbilical artery-pulsatility index (UA-PI) > 95th percentile.

Exclusion criteria included fetal structural or chromosomal abnormalities, maternal heart disease, maternal or fetal conditions required urgent delivery such as preeclampsia with severe features, intra-amniotic infection, fetal compromise, or women in active labor.

A detailed history was obtained from each patient, followed by an estimation of gestational age based on a reliable last menstrual cycle or early dating ultrasound scan, followed by ultrasonography and Doppler examination to assess the following: gestational age, fetal viability, EFW, exclusion of congenital anomalies, check amniotic fluid volume.

The cohort was randomly divided into two groups through a computer-generated randomization sheet using MedCalc version 13. Patients were allocated to either Group A (intervention) or Group B(control) in a 1:1 ratio. Forty opaque sealed envelopes numbered from 1 to 40, each contain a card showing the group assignment, were prepared and randomly assigned to patients. The drug administration was performed by a resident. By employing computer-generated randomization techniques, we aimed to avoid selection bias and enhance the validity of the findings.

Group A received Nitroderm TTS ^®^ transdermal patch, 5 mg (nitroglycerin 5 mg) (Novartis company) applied to a clean, non-hairy, dry area of intact skin on the trunk or upper arm with holding the patch in position for 10–20 s with the palm of the hand. It is utilized for 12 h daily to prevent tolerance from building up with PVE in the form of oral fluid intake (2.5 L per day of water) [14]. Group B did not receive neither nitroglycerine nor PVE.

Fetal growth will be followed up after 2 weeks for EFW (AC, BPD, HC, FL) with assessment of the amniotic fluid index. While frequency of assessment of the fetal wellbeing will depends on the severity of FGR and UA Doppler assessment, minimum weekly or 2–3 times weekly when there are UA Doppler abnormalities. Assessment of fetal wellbeing was performed by non-stress test (NST) or biophysical profile (BPP) [1]. Delivery was considered if fetal surveillance tests indicated fetal compromise after a course of corticosteroid.

The primary endpoint of the study, evaluated after 2 weeks from the initiation of treatment (or until delivery if earlier), focused on evaluating fetal growth through EFW and AC, which served as the primary outcome. Secondary outcomes encompassed the evaluation of the amniotic fluid index (AFI), umbilical artery Doppler indices (Resistant index and pulsatility index), as well as monitoring maternal vital signs (blood pressure and pulse) and potential side effects (headache, palpitation, postural hypotension). Continued medications were advised in case of improvement of fetal growth and Doppler indices until delivery, with additional measurements taken at delivery, including estimated gestational age (GA), interval to delivery, route of delivery, birth weight, and APGAR score assessment.

### Sample size calculation

The required sample size has been calculated using the Power Analysis and Sample Size (PASS^©^,) software version 11.0.10 (NCSS, LLC, Keysville, Utah, USA). A previous study showed birth weight in grams was significantly higher in those women randomized to receive nitric oxide + plasma volume expansion vs the control group (pregnant women not receiving nitric oxide nor plasma volume expansion) (1283 ± 366 gm vs 750 ± 223 gm) [22]. Calculation according to these values produced a minimal sample size of 12 cases. So, it is estimated that a sample size of 40 patients would achieve a power of 95% (type 2 error) to detect equivalence of both observations. These calculations used two, one-sided Z tests and the significance of the tests is targeted at the alpha = 0.05 level (confidence level = 95%).

### Statistical analysis

Analysis is to be performed using SPSS for windows v20.0, Data to be presented in terms of range, mean and standard deviation (for numeric parametric variables); range, median and inter-quartile range (for numeric non-parametric variables); or number and percentage (for categorical variables). Difference between two independent groups is to be analyzed using independent student’s t test as well as the mean difference and its 95% CI (for numeric parametric variables); or Chi-squared test as well as the risk ratio and its 95% CI (for categorical variables). Binary logistic regression analysis is to be performed for estimating the association between good/poor response and the measured variables ROC curves are to be constructed for estimating the validity of measured variables as predictors of good or poor response validity is to be presented in terms of sensitivity, specificity, positive and negative predictive values and their corresponding 95% Cis significance level is set at 0.05.

## Results

### Consort flow diagram

#### IUFD: Intra uterine fetal demise; AEDF: Absent end diastolic flow; REDF: Reversed end diastolic flow; BW: Birth weight

A total of 52 pregnant women diagnosed with early-onset FGR were initially recruited. Out of these, four did not meet the inclusion criteria due to associated congenital anomalies, three declined to participate, and five were lost to follow-up. The remaining 40 pregnant women were enrolled in the trial, 20 in the intervention Group A, and 20 in the control Group B.

Patient characteristics and variables at enrollment are summarized in Table 1. There were no significant difference regarding age, BMI, parity, and gestational age between the two groups. In addition, maternal hemodynamics and ultrasound fetal biometry were comparable between both groups, with all P values are greater than 0.05.

**Table 1:** Maternal characteristics and variables at enrollment

Variable	Group A	Group B	*P* value
Age (year)	27.85 ± 4.83	27.9 ± 4.56	0.973^*^ NS
BMI (kg/m^2^)	25.77 ± 2.16	24.63 ± 3.11	0.186^*^ NS
Parity (number)	1.75 ± 1.16	1.95 ± 1.09	0.579^*^ NS
GA at enrollment (weeks)	30.25 ± 1.51	30.15 ± 1.49	0.834^*^ NS
EFW at enrollment (gm)	1399.81 ± 241.24	1369.99 ± 209.78	0.678^*^ NS
EFW centile at enrollment	7.77 ± 1.72	7.7 ± 1.46	0.890^*^ NS
AC at enrollment (mm)	251.35 ± 14.64	249.78 ± 12.83	0.719^*^ NS
HC at enrollment (mm)	274.64 ± 12.37	273.46 ± 11	0.750^*^ NS
BPD at enrollment (mm)	74.02 ± 3.78	73.39 ± 3.19	0.570^*^ NS
FL at enrollment (mm)	55.28 ± 3.33	54.88 ± 2.97	0.695^*^ NS
AFI at enrollment	7.6 ± 2.97	7.87 ± 3.27	0.782^*^ NS
UA RI at enrollment	0.8 ± 0.07	0.79 ± 0.06	0.761^*^ NS
UA PI at enrollment	1.31 ± 0.1	1.25 ± 0.12	0.113^*^ NS
Systolic BP at enrollment (mmHg)	148.3 ± 18.03	146.3 ± 15.89	0.711^*^ NS
Diastolic BP at enrollment (mmHg)	92 ± 12.94	94.1 ± 10	0.569^*^ NS
Pulse at enrollment (bpm)	78.05 ± 6.19	77.9 ± 6.26	0.939^*^ NS

*All values (continuous quantitative data) are given as mean* ± *SD*

*NS non-significant*

*t test for normally distributed data

*P value* < *0.05 significant*

After a two-week period, Group A showed significant improvements in EFW, AC, HC, BPD, FL, amniotic fluid index, and UA Doppler PI with P values 0.032, 0.041, 0.010, 0.011, 0.038, 0.037, 0.042, respectively as shown in Table 2. However, maternal hemodynamics such as systolic and diastolic blood pressure and pulse did not show statistically differences between the two groups.

**Table 2:** Maternal hemodynamics and ultrasound fetal biometry of both groups after 2-week period

Variable	Group A	Group B	*P* value
EFW after 2 weeks. (gm)	1857.7 ± 274.5	1676.075 ± 240.91	0.032^*^ s
EFW centile after 2 weeks	11.81 ± 5.4	8.68 ± 3.8	0.040^*^ s
AC after 2 weeks. (mm)	274.86 ± 15.29	265.2 ± 13.45	0.041^*^ s
HC after 2 weeks. (mm)	294.92 ± 11.35	285.80 ± 9.96	0.010^*^ s
BPD after 2 weeks. (mm)	80.18 ± 3.53	77.4 ± 3.07	0.011^*^ s
FL after 2 weeks. (mm)	60.65 ± 3.42	58.5 ± 2.92	0.038^*^ s
AFI after 2 weeks. (cm)	12.45 ± 4.27	10.0 ± 3.0	0.037^*^ s
UA RI after 2 weeks	0.71 ± 0.13	0.8 ± 0.12	0.049^*^ s
UA PI after 2 weeks	1.13 ± 0.28	1.33 ± 0.3	0.042^*^ s
Systolic BP after 2 weeks. (mmHg)	143.05 ± 18.14	146.85 ± 14.08	0.463^*^ NS
Diastolic BP after 2 weeks. (mmHg)	86.85 ± 8.83	87.8 ± 10.7	0.761^*^ NS
Pulse after 2 wk. (bpm)	77.65 ± 7.05	75.6 ± 6.27	0.337^*^ NS

*All values (continuous quantitative data) are given as mean* ± *SD*

*NS non-significant, S significant*

*t test for normally distributed data

*P value* < *0.05 significant*

Table 3 demonstrated that Group A had more favorable outcomes in terms of later gestational age at delivery, increased delivery interval, neonatal birth weight, APGAR scores, lower NICU admission rates, and less deterioration in Doppler indices, with statistically significant P values of 0.018, 0.048,0.041,0.003, 0.01, 0.026, and 0.026, respectively. Conversely, there were no statistically significant difference regarding the mode of delivery, incidence of IUFD, fetal distress, and neonatal death with P values 0.16, 0.151, 0.102, and 0.375, respectively.

**Table 3:** Fetal, neonatal, and delivery outcomes

Variable	Group A	Group B	*P* value
Estimated GA at delivery (weeks)^1^	36.13 ± 1.98	34.56 ± 2.04	0.018^*^ s
Interval to delivery (days)^1^	37.8 ± 19.28	27.1 ± 13.3	0.048^*^ s
Route of delivery^2^			
CS	12 (60%)	16(80%)	0.16^#^
VD	8 (40%)	4(20%)	NS
Birth weight (gm)^1^	2497.143 ± 515	2149.8 ± 524.46	0.041^*^ S
Birth weight centile at delivery^1^	20.55 ± 13.16	12.5 ± 10.5	0.038^*^ S
APGAR score at 1 min^1^	6.65 ± 2.13	4.3 ± 2.55	0.003^*^ S
APGAR score at 5 min^1^	8.35 ± 3.32	5.95 ± 3.3	0.011^*^ S
NICU admission^2^			
Yes	7 (35%)	14(70%)	0.026^#^
No	13(65%)	6(30%)	S
IUFD^2^			
Yes	1(5%)	4 (20%)	0.151^#^
No	19(95%)	16 (80%)	NS
Fetal distress^2^			
Yes	5(25%)	10 (50%)	0.102^#^
No	15(75%)	10 (50%)	NS
Deterioration of doppler indices requiring delivery^2^			
Yes	6(30%)	13 (65%)	0.026^#^
No	14(70%)	7 (35%)	S
Neonatal death^2^			
Yes	2(10%)	4 (20%)	0.375^#^
No	18(90%)	16 (80%)	NS

*Values*^*1*^* (continuous quantitative data) are given as mean* ± *SD. Values*^*2*^* (numerical data) are given as numbers (percentage)*

*t test for normally distributed data

^*#*^*Chi-Square test to determine P value*

The follow-up is detailed in the CONSORT flow diagram. In Group A, three out of the 20 pregnant women delivered earlier than two weeks due to intrauterine fetal demise (IUFD), deterioration of umbilical artery Doppler (REDF), or fetal distress. Among the remaining 17 women, 14 showed improvement and continued to receive the NO patch until delivery, while three did not improve and had the NO patch discontinued. In Group B, five patients delivered early, with four cases of IUFD and one case of Doppler deterioration (AEDF). Of the remaining 15 patients, eight showed improvement, while seven did not, with three of the seven experiencing neonatal deaths.

The neonatal birth weight is summarized in Table 4, classified based on whether it falls above or below the 10th centile. This classification is then compared to the previous response during the antenatal period to determine the trend of either improvement or deterioration in EFW. The results were statistically significant regarding the percentage of neonatal birth weight percentiles in Group A, while Group B did not show significant results. In Group A, 92% of the women who had improved during pregnancy maintained birth weights above the 10th percentile at delivery. Similarly, in Group B, 87% continued to have birth weights above the 10th percentile. It is important to note that when comparing the two groups, the results were statistically significant in favor of Group A, indicating greater antenatal enhancement in fetal weight and higher postnatal neonatal birth weights above the 10th percentile.

**Table 4:** Birth weight centile at delivery and comparing the results to their previous responses during pregnancy in Groups A and B

**Group A Birth weight centile at delivery**			
	**< 10th centile**	**> 10th centile**	*P* value
Percentage of neonates	6/20 (30%)	14/20 (70%)	*P* = 0.0125^#^ S
Percentage who previously improved during pregnancy	1/6 (16.6%)	13/14 (92.85%)	*P* = 0.0009^#^ S
Percentage who previously deteriorate during pregnancy	5/6 (83.33%)	1/14 (7.14%)	P = 0.0009^#^ S
**Group B Birth weight centile at delivery**			
	**< 10th centile**	**> 10th centile**	*P* value
Percentage of neonates	12/20 (60%)	8/20 (40%)	*P* = 0.211^#^ NS
Percentage who previously improved during pregnancy	1/12 (8.33%)	7/8 (87.5%)	*P* = 0.006^#^ S
Percentage who previously deteriorate during pregnancy	11/12 (91.66%)	1/8 (12.5%)	*P* = 0.006^#^ S
	**Group A**	**Group B**	*P* value
Percentage who improved antenatal based on EFW centile	14/20 (70%)	8/20 (40%)	*P* = 0.0286^#^ S
Percentage of neonatal birth weight > 10th centile	14/20 (70%)	8/20 (40%)	*P* = 0.0286^#^ S

^*#*^*Chi-Square test to determine P value*

*Statistically significant at P* < *0.05*

Maternal side effects, summarized in Table 5, indicated that side effects such as headache, palpitation, and postural hypotension were slightly more common in Group A compared to Group B, but none of these differences were statistically significance, with all P values are greater than 0.05.

**Table 5:** Maternal side effects

Variable	Group A	Group B	*P* value
Headaches	4 (20%)	2 (10%)	0.661^# NS^
Palpitation	2 (10%)	0 (0%)	0.487^# NS^
Postural hypotension	4 (20%)	2 (10%)	0.661^# NS^

*Values (numerical data) are given as numbers (percentage)*

*NS non-significant*

^*#*^*Chi-Square test to determine P value*

*P value* < *0.05 significant*

## Discussion

Early-onset FGR is linked to abnormal placentation, leading to increased hypoxia and cardiovascular adaptation, consequently carrying a greater risk of adverse perinatal mortality and morbidity [1]. Currently, there are no effective treatments available. Some evidence suggests that certain medications may enhance uteroplacental blood flow, potentially improving fetal growth and neonatal outcomes. These include NO donors (Such as Nitroglycerine, L-arginine, Isosorbide mononitrate, and Pentaerithrityl tetranitrae PETN) and Sildenafil.

The multicenter, randomized, placebo-controlled double-blind trial, STRIDER, investigated maternal sildenafil for severe FGR but found no significant benefits in prolonging pregnancy or improving outcomes. The Dutch arm of the trial was halted early due to higher than expected neonatal mortality and rates of persistent pulmonary hypertension of the newborn (PPHN). In response to these findings, a physician alert was issued advising against prescribing sildenafil for FGR, leading to the early termination of the Canadian trial [16, 17].

There are conflicting results regarding PETN; some studies demonstrate a positive impact in pregnancies complicated by impaired placental perfusion [18, 19], while others show no effect on FGR or perinatal mortality [20, 21].

The vasodilatory effect of NO on placental vascular tone shows a promising effect, but maternal side effects limit its effectiveness. Consequently, our study aimed to evaluate the effect of combining NO donors, through nitroglycerine transdermal patch – which is readily available—with PVE on pregnancies complicated by early-onset FGR.

Our study demonstrates a significant improvement in fetal growth, amniotic fluid volume, Doppler indices, and perinatal outcomes in the intervention group compared to the control group. In addition, maternal side effects were well tolerated with no significant differences between the two groups.

The findings of our study are consistent with previous research indicating the potential benefits of NO donors in enhancing uteroplacental blood flow and fetal growth in cases of early-onset FGR. After two weeks of treatment, Group A demonstrated significant improvements in key growth parameters such as EFW, AC, HC, BPD, FL, and amniotic fluid index, compared to Group B. Women in Group A who displayed improvements in fetal growth parameters decided to continue NO donor treatment until delivery, considering the known poor prognosis of the early-onset FGR. Continuously monitoring them until delivery also revealed a significant enhancement in neonatal birth weight. That aligns with previous studies which suggested that NO donors can enhance vasodilation in the maternal systemic circulation, which, in turn, improves blood flow to the placenta, thus fostering better fetal growth outcomes [13–15].

Furthermore, Tiralongo et al., [14], and Valensise et al., [22], reported that pregnancies treated with NO donors exhibited higher EFW centiles at diagnosis and improved birth weights compared to untreated controls [14]. The improvement in fetal growth metrics can be attributed to the dual action of NO on maternal hemodynamics and placental blood flow, as well as the synergistic effect of PVE in increasing intravascular volume and cardiac output [22].

Moreover, Di Iorio et al. [23], found that NO donor therapy in FGR patients led to a significant reduction in umbilical artery resistance index (RI), a finding that was corroborated in our study by the trend towards lower umbilical artery RI in Group A. This suggests that NO donors can improve placental blood flow by reducing the resistance in umbilical arteries, thereby enhancing oxygen and nutrient delivery to the fetus [14, 22]. Controversy, the study by Lees et al. [24] noted a lack of significant effect on uterine artery RI with low dose NO donor patches, which may highlight the importance of dosage and the need for fluid management to optimize the therapeutic effects of NO donors.

Furthermore, our study contributes to the growing evidence suggesting that extending gestation in FGR pregnancies can significantly reduce the risk of adverse neonatal outcomes. Bilardo et al. [25] demonstrated that for every additional week of gestation, there is a 50% reduction in the incidence of adverse outcomes, a finding that consistent with the prolonged gestation observed in Group A, where the mean latency to delivery was significantly longer compared to Group B. This extended gestation likely allowed for further fetal growth and maturation, reducing the likelihood of severe neonatal morbidity and mortality.

The NO donor likely extended pregnancies by improving uteroplacental blood flow and exhibiting a tocolytic effect; however, a definitive link to reduced preterm labor risk could not be established. In Group B, initial premature deliveries were primarily due to IUFD or worsening umbilical artery Doppler indices. The same finding was reported in Group A among those who deteriorated and delivered within the first 2 weeks. Furthermore, individuals in Group B who continued their pregnancies after two weeks faced premature deliveries linked to fetal distress or Doppler deterioration. Among participants in Group A who did not show improvements in fetal growth parameters had the administration of the NO patch discontinued. In these cases, fetal distress, worsening Doppler results, and neonatal birth weights below the 10th percentile were observed, leading to delivery.

In terms of neonatal outcomes, Group A had significantly better birth weights, higher APGAR scores, and lower rates of NICU admission. These outcomes are indicative of improved fetal health and are likely a direct consequence of the enhanced placental perfusion and prolonged gestation achieved through NO donor and PVE therapy. Tiralongo et al.‘s study supports these findings, showing that NO donor treatment led to higher birth weights and better birth-weight centiles compared to untreated controls [14].

However, certain outcomes, such as mode of delivery, IUFD, and neonatal death, were higher among Group B, but the results did not reach statistical significance, potentially due to the study’ limited sample size. This aligns with the challenges faced by Lees et al. [24] and Melchiorre et al. [26], where small sample sizes and the observational nature of their studies limited the ability to draw firm conclusions regarding perinatal outcomes. Future studies with larger populations are necessary to validate these findings and provide more robust data on the efficacy of NO donor and PVE therapy in FGR.

The observed side effects in Group A, including headaches, palpitations, and postural hypotension, were more common than in Group B but did not reach statistical significance. This suggests that the combined therapy of NO donors and PVE is relatively safe and does not substantially increase the risk of these specific adverse effects, a conclusion supported by the broader literature on NO donor safety in pregnancy [27].

Considering patient response to NO donors, the concurrent use of antihypertensive medications, often linked to hypertension-associated FGR, was noted without significant side effects requiring discontinuation. The 12 h transdermal patch application, in conjunction with PVE, likely helped in managing adverse effects, underscoring the safety and tolerability of the combined therapy.

A key strength of our study is the combined use of NO donor with PVE, which mitigates maternal side effects, along with the comprehensive assessment of maternal and fetal outcomes. However, the limitations include a small sample size, and the fact that the included pregnant women had forward end diastolic flow in umbilical artery Doppler (indicating increased resistance), which may limit the generalizability of the results. Although the target population in the study used in power calculation was pregnant women with gestational hypertension — unrelated to our study— both studies focused on fetuses with growth restriction. The study utilized for the power calculation demonstrated improved fetal growth with the use of nitric oxide donors and PVE, prompting us to employ it for calculating the sample size and emphasizing the surrogate outcome that correlates with the clinical endpoint, which is fetal weight as a primary outcome and neonatal weight as a secondary outcome, instead of the Doppler changes.

Despite these limitations, the study provides valuable insights into the potential benefits of NO donor and PVE therapy in managing FGR. The observed enhancement in fetal growth without a significant maternal side effect, suggest that this combined therapy could offer a promising approach to mitigating the adverse effects of FGR. However, larger, randomized controlled trials with FGR associated with severe Doppler changes are needed to confirm these findings and to establish standardized protocols for the use of NO donors and PVE in this context. In addition, a larger sample size is required to analyze the subgroup that did not exhibit improvement with treatment and its potential connection to the development of hypertensive disorders during pregnancy.

## Conclusion

In conclusion, this study supports the hypothesis that the combined use of NO donors and PVE in pregnancies complicated by early-onset FGR can significantly enhance fetal growth and prolong gestation. These findings contribute to the growing evidence that NO donor therapy, when appropriately combined with fluid management, can offer substantial benefits in the management of FGR. However, further research is necessary to validate these results and to refine the therapeutic approach for optimal outcomes in affected pregnancies.

## Data Availability

The raw data for the analysis are available.
